# Supplementary material for: Lead acetate–based test strip method for rapid and quantitative detection of residual sulfur dioxide in Chinese herbal medicines
Source: PLoS One. 2024 Nov 12;19(11):e0310628. doi: 10.1371/journal.pone.0310628 (PMC11556703; doi:10.1371/journal.pone.0310628)
Supplement: Attachment — Submitted filename: 1. Response to Reviewers.docx [file pone.0310628.s001.docx]

Dear Editor and reviews：

Thanks for the fair, encouraging and constructive comments from you and the reviewers. We learned a lot. After carefully studying the comments and your advice, we have made corresponding changes. We have revised the paper according to your comments.

We have taken the reviewer's comments seriously and have made the necessary revisions to our manuscript to address the concerns raised. We believe that our responses have strengthened the paper and hope that the revised version meets the journal's standards.

All of the authors approved the submitted version (and any substantially modified version that involves the author’s contribution to the study) and All of the authors agrees to be personally accountable for the author’s contributions AND to ensure that questions related to the accuracy or integrity of any part of the work are appropriately investigated, resolved, and the resolution documented in the literature

This work was supported by the Key Research and development program of Shaanxi Province(2024SF-YBXM-081), Shaanxi provincial People's Hospital Science and Technology Personnel Support Program(No.2021LJ-02), Key Research and development program of Shaanxi Province(No.2022KWZ-20), Shaanxi provincial People's Hospital Science and Technology Development Incubation Fund（2023YJ-22）Shaanxi Provincial health and Medical Research Innovation Capacity Enhancement Plan(2024PT-01), Shaanxi Province Innovation Capability Support Program Project (S2024-ZC-TD-0127), Shaanxi Provincial health high-level talent cultivation program, the Shaanxi Special Support Plan for High Level Talents. The funders had no role in study design, data collection and analysis, decision to publish, or preparation of the manuscript.

Please find the revised manuscript along with the detailed responses to the reviewer's comments. We kindly request that the editor please forward the file to the reviewer for further consideration. our revised manuscript include the following items:

'Response to Reviewers'.

'Revised Manuscript with Track Changes'.

'Revised Manuscript'.

Thank you for your attention to this matter, and we look forward to the journal's decision on our submission.

Penghua Zhao

Thanks of additional editor comments, and We did not multiple submissions for one draft, this is our original article. We corrected all the comments above point to point. See the corrected article for details

The Figure 1 in our submission contain copyrighted images. Thank you for your insightful comments. We appreciate your guidance and understand the importance of adhering to the CC BY4.0 license requirements. The figure in this paper is similar but not identical to the original image for illustrative purposes only, we have clearly indicated this in the figure caption, including the source information as required.

We assure you that we will address these matters with the utmost diligence and professionalism. Thank you for your understanding and support throughout the peer-review process.

We revised on the reviewer's suggestion regarding the inclusion of tables within the main text of our manuscript and the retention of supplementary tables as separate "supporting information" files.

We outlined every change made in response to the comments and provide suitable explanation for the comments not addressed. The main revisions are listed as follows:

Reviewer #1:

1. Please reorganize the abstract part. Background, Methods, Results and Conclusions should not be indicated.

We were happy to reorganize the abstract, based on helpful comments from the reviewers. And we have reorganized the abstract part without Background, Methods, Results and Conclusions. The new abstract is as follows:

To prevent the residual sulfur dioxide in Chinese herbal medicines (CHM) caused by sulfur fumigation, which may lead to severe health issues, there is an urgent need for a rapid and quantitative detection technique. Sodium borohydride was used as a reducing agent to convert sulfur dioxide into hydrogen sulfide, which was then detected using lead acetate test strip. An accurate testing apparatus was designed, consisting of reaction bottle cap, reaction bottle, lead acetate test strip, and sulfur dioxide detector. The effect of different reaction variables on detection, including reductant quality, pH of initial media, reaction time, lead acetate concentration, and membrane type was investigated. The optimal conditions were determined by orthogonal experiments. The reaction membrane type and lead acetate concentration on the membrane were optimized to enhance detection accuracy. Standardized gray cards were fabricated and used to calibrate the detector. The detection system demonstrated an exceptional linear correlation (r^2^=0.9992), with a linear detection range of 0–750 mg·kg^−1^. The colored substances and sulfur-containing substances within the matrix of CHM did not affect the detection results. Therefore, the detection method exhibited superior accuracy and stability. The proposed technique proved to be swift, reliable, and provides a straightforward and convenient approach for the quantitative determination of sulfur dioxide in CHMs. The results of this work may provide insights into the development of test strips for quantitative detection.

2. Please rewrite the keyword ‘Quantitative’.

Thanks for your comments. I rewrited the keyword ‘Quantitative’ to ‘Quantitative detection’

3. The section 2. Materials and methods need to be reorganized. There are too many subtitles which make experimental details unclear.

We revised. We reorganized the section 2. Materials and methods. Thank you for your feedback. It is important to provide clear and detailed experimental details to ensure readability. I have removed the subtitles and reorganized the Materials and methods.

4. Please reorganize the results part by reducing subtitles and extract the main results followed by significant biological explication.

We have reorganized the results section by reducing subtitles and extracting the main results with significant biological explanations.

5. Please add scale in Figure 1.

We agree with this suggestion and have added scale in Figure 1.

6. Please add error bar in the experimental curves.

We have added the error bar in the experimental curves, based on helpful comments from the reviewers.

7. The figutes are not good presented.

We've improved the way that data presented and edited the text based on helpful comments from the reviewers.

8. The table 2 is not good enough.

We thank the reviewer for pointing out this issue. Table 2 is a typical table in orthogonal

studies, which can clearly show which level of factors contribute to the test results. Probably because I didn't make the meaning of the table clear.

In Table 2, the first column of each row represents the serial number of the experiment, the second to fourth columns represent the conditions used for the experiment, and the fifth column represents the experiment's results under the above conditions. For example, 1 in the first column of the second row indicates this is the experiment 1. And 1 in the second column indicates that in detection experiment 1, the condition of factor Fa is Level 1, in which the NABH4 concentration is 0.5 mm; 1 in the third column of the second row indicates that, in detection experiment 1, the condition for factor Fb is Level 1, that is, the experimental time is 1 min; 1 in the fourth column indicates that, in detection experiment 1, the Factor Fc condition is Level 1, which means the experimental Initial pH condition is 5.0; in the column 5, 77.3 indicate that under these conditions, the sulfur dioxide recovery rate of this test is 73.3%.

Reviewer #2: In section 3.2. Preparation of standard grayscale cards, teststrips with a lead acetate concentration of 15% were prepared. However, in section 3.1.2.1. and Figure 2a, lead acetate with concentration of 20% showed better linear pattern of the detected gray value.

Thank you for your observation. To clarify, in Section 3.1.2.1 and Figure2a, we observed a consistent linear pattern in the detected gray values for lead acetate solutions with concentrations of 15% and 20%. And there is no statistical difference between 15% and 20%, so the results are good, to save raw materials I choose 15%.

In section 3.4.2.1. Precision testing, SO2 residual concentration for the three herbs was not clearly addressed in the method part.

We appreciate your attention to detail. we add SO_2_ residual concentration for the three herbs in section 3.4.2.1.

The colors of the line on Figure 2a can’t correspond to the caption beside it.

Thank you for observing the color coding of the lines in Figure 2a. You are correct; there was an inconsistency between the figure and its caption. I have made the necessary modifications to ensure that the colors of the lines now correspond accurately to the legend provided beside the figure. And figure 2 has been redrawn.

Reviewer #3:

1. In “Introduction” section:

Why don't you refer to the latest edition of Chinese Pharmacopoeia (2020)?

Thank you for your suggestion to reference the most recent edition of the Chinese Pharmacopoeia (2020). It is true that the latest quotations can reflect the present problems, and I had added the present situation in my article. In the introduction section, we refer to the 2010 edition of the Pharmacopoeia to show that as early as 2010, sulfur dioxide abuse was already a serious problem.

In addition to the mentioned common detection methods in addition to acid-base titration, ion chromatography, gas chromatography, fluorescence derivatization, colorimetry, etc., there are also many methods for the rapid detection of sulfur dioxide residue in Chinese herbal medicine. Please provide an overview of the latest research to highlight the need for the detection methods in this study.

Thank you for your insightful query regarding the detection methods for sulfur dioxide residue in Chinese herbal medicine. Based on your comments, We have checked and overviewed the latest research to highlight the need for the detection methods and I checked and cited 1.(*Lan W, Rao Y, Zhao X, et al. Rapid visual detection of sulfur dioxide residues in food using acid-sensitive CdTe quantum dots-loaded alginate hydrogel beads. Food Chem. 2024;446:138791. doi:10.1016/j.foodchem.2024.138791*) which is sers-based detection methods and 2(*Fan B, Wang Y, Li Z, et al. Si@Ag@PEI substrate-based SERS sensor for rapid detection of illegally adulterated sulfur dioxide in traditional Chinese medicine. Talanta. 2022;238(Pt 1):122988. doi:10.1016/j.talanta.2021.122988*) which is quantum dots -based detection methods

2. In “Methods” section:Please attach the chemical reaction formula.

Thank you for your instruction to include the chemical reaction formulas in the "Methods" section of our manuscript. We will gladly incorporate these formulas as suggested. We appreciate the reviewers' insightful comments that have guided us in making these improvements. We will ensure that the reaction formulas are accurately and clearly presented, enhancing the comprehensiveness of our methodology description. And will be happy to added the chemical reaction formula based on helpful comments. The reaction formula as follow:

What was the reason for choosing sodium borohydride (NaBH₄) as the reducing agent? The reaction between sulfur dioxide or sulfite and sodium borohydride is not a common chemical reaction because their chemical properties do not directly promote such reactions under normal conditions. However, under certain conditions, the two compounds may react, but this is rare and the reaction conditions are usually harsh. What reaction conditions were used in this study to facilitate the reaction? This point is not seen in the article at present!

In response to your query regarding the selection of sodium borohydride (NaBH₄) as a reducing agent in our study, we would like to clarify that NaBH₄ is a commonly used reducing agent in the coal chemical industry for the removal of sulfur from sulfur-containing carbonaceous materials. Its selection was based on several compelling properties:

Reductivity: NaBH₄ exhibits strong reducing capabilities, making it effective for sulfur dioxide (SO₂) and sulfite (SO₃²⁻) reduction under room temperature conditions.

Temperature Stability: The compound remains stable within a wide temperature range of -50°C to170°C, which is beneficial for controlling reaction conditions.

Air Stability: NaBH₄ is air-stable and does not pose an explosion risk when stored under appropriate conditions.

Shelf Life: Under the correct storage parameters, NaBH₄ has a shelf life of over24 months, making it a versatile initiator.

The reaction between SO₂/SO₃²⁻ and NaBH₄ is under acidic conditions due to their inherent chemical properties. We will ensure that these conditions are thoroughly described and justified in the revised submission to address the concerns raised by the reviewer. By adding hydrochloric acid to the reaction solution, the pH of the solution is below 5, which satisfies the sodium borohydride reduction of sulfur to hydrogen sulfide, and the amount of hydrochloric acid added in the detection system was 2 ml of 2 mol L HCl, as reported in section 2.2.2.

Why not use real Chinese medicine for research? In this study, what is the role of Chinese herbal medicine extract in it? Is it just a simulation? The real detection method will carry out pre-extraction treatment of Chinese medicinal materials, and the pre-treatment steps will also affect the reaction conditions. Therefore, the use of Chinese medicinal materials for real detection is an essential part of the establishment of the new methodology!

Thank you for your feedback. In our research, the purpose was to establish a method for detecting sulfur dioxide based on lead acetate detection paper. For the extraction solution used in the study, we chose it because it is simple in composition and conducive to analyzing and detecting reaction conditions. In our future research, we agree that it is essential to conduct actual sample testing to investigate the applicability of the method. We would like to express our gratitude for your careful.

It is suggested that a batch of Chinese medicinal materials should be simultaneously tested by other known mature methods, such as acid-base titration in Chinese Pharmacopoeia, in order to demonstrate the accuracy of the detection results of this method.

We appreciate the suggestion to validate our newly developed Lead acetate-based test strip method for the rapid and quantitative detection of residual sulfur dioxide in Chinese herbal medicines through comparison with established methods such as acid-base titration as outlined in the Chinese Pharmacopoeia. However, We wish to emphasize that the primary objective of our study is to introduce and optimize this novel method. Introducing a comparison with other methods might detract from the core focus of our article, which is to showcase the development and refinement of our test strip approach.

We are committed to providing robust evidence to support the accuracy and reliability of our method. Instead of diverting the focus of our paper, we have increased a section named application verification that highlights the comparison of our method with conventional techniques as a separate, supporting study. This approach would allow us to maintain the integrity of our main research theme while providing valuable comparative data to this study. We hope this compromise addresses the editor's concerns and we are open to further discussion on this matter.

3. In “3.1.1”:In this part, the best reaction time was determined to be 10 min. Why was 15 min used in 3.1.2.1. to detect the influence of different concentrations of lead acetate on the detection accuracy?

Thank you for your astute observation regarding the discrepancy in the reaction time used in section3.1.2.1. to assess the influence of different concentrations of lead acetate on detection accuracy.

In the initial stages of our research, the study on the membranes was conducted at a factor level without precise data validation. At that time, the chosen reaction time of 15 minutes was an estimate based on extensive experience, which we believed ensured sufficient reaction time for accurate results. However, subsequent experiments have shown that a reaction time of 10 minutes is sufficient for accurate detection.

In the interest of maintaining consistency and objectivity in our manuscript, we have made the decision to revise the reaction time to 10 minutes, as this aligns more closely with our current empirical findings. We appreciate your guidance in highlighting this issue, and we believe that this adjustment better represents the accurate and reliable outcomes of our research.

We hope this explanation clarifies our approach, and we are grateful for the opportunity to refine our manuscript.

4. In “3.1.2.1”:

In this part, CN95 membrane was used to detect the influence of different concentrations of lead acetate on the detection accuracy. However, the best experimental membrane type was determined as CN 140 in 3.1.2.2. Please explain the different membrane types of the two parts!

Should the optimal membrane type be determined first, and then the influence of the concentration of lead acetate under the optimal membrane type on the detection results be tested?

Thank you for your inquiry regarding the use of different membrane types in the study of the influence of lead acetate concentrations on detection accuracy.

Our initial selection of the CN95 membrane was based on the understanding that the response of the membrane to lead acetate concentrations would follow a consistent trend, with the concentration of lead acetate being a more significant factor affecting the detection results. Subsequently, after thorough evaluation, we identified the CN140 membrane as the optimal choice for quantitative detection under the determined optimal lead acetate concentration.

We appreciate your guidance and will revise our methodology accordingly to reflect this more logical sequence of experimentation.

5. In “Discussion” section:

There is no discussion section in this paper. Please supplement the latest progress of relevant technology in the current research field, compare it with this study, and discuss the limitations and future research direction of this technology!

Thank you for your thorough review and feedback on our manuscript. I fully agree with your suggestion to include a discussion section, and we are pleased to provide the requested information.

Firstly, regarding the lack of discussion section, I apologize for the oversight. We have added a new section to the end of the paper, discussing the latest progress of relevant technology in the current research field, comparing it with our study, and discussing the limitations and future research direction of this technology.

Regarding the limitations of our technology, we acknowledge that the membrane coloration linear range is limited. However, we have also identified potential future research directions that could address this limitation, including exploring new coloration methods and developing more sensitive detection methods.

Finally, the future development of this technology should focus on automation and high-throughput detection methods. We believe that these advancements would greatly improve the practicality and efficiency of this technique.

We appreciate your time and feedback, and we are committed to improving our manuscript accordingly. Thank you again for your valuable advise.

Reviewer #4:

1. Why the membrane type has effect on detection accuracy? The authors should address it in the context.

We appreciate the opportunity to respond to the reviewer's comments. With regard to the question regarding the membrane type's effect on detection accuracy, it is important to note that differences in the manufacturing process or material composition of different brands of nitrocellulose membrane can have an impact on color presentation.

For instance, thicker membranes may reflect more light, resulting in a more vivid color, while higher-density membranes may absorb more light, leading to a deeper color. Additionally, smoother surfaces may result in more uniform reflection of light, leading to a more uniform and pure color. The specific chemical composition of the membrane may also affect the absorption and reflection of light, thereby influencing color presentation.And we had address it in the context.

2. The language need to be improved and polished.

Thanks for your comments. We have performed language editing of this manuscript at the recommended non-native English language editing company and provided proof.

Thanks to all reviewers for their comments on our manuscript, we have made point-to-point revisions, please check.
